# Development and comparison of cross-linking and non-crosslinking probe-gold nanoparticle hybridization assays for direct detection of unamplified bovine viral diarrhea virus-RNA

**DOI:** 10.1186/s12896-021-00691-w

**Published:** 2021-04-23

**Authors:** Zahra Heidari, Seyedeh Elham Rezatofighi, Saadat Rastegarzadeh

**Affiliations:** 1 Department of Biology, Faculty of Science, Shahid Chamran University of Ahvaz, Ahvaz, 6135743135 Iran; 2 Department of Chemistry, Faculty of Science, Shahid Chamran University of Ahvaz, Ahvaz, Iran

**Keywords:** Bovine viral diarrhea virus, Gold nanoparticles, Cross-linking, Non-crosslinking, Hybridization assay

## Abstract

**Background:**

Bovine viral diarrhea virus (BVDV) is a major economic disease that has been spread in most countries. In addition to vaccination, one of the main ways to control the disease and prevent it from spreading is to detect and cull infected animals, especially those with persistent infection (PI). We developed and compared two colorimetric biosensor assays based on probe-modified gold nanoparticles (AuNPs) to detect BVDV. Specific probes were designed to detect the 5′ untranslated region of BVDV-RNA. The thiolated probes were immobilized on the surface of the AuNPs. Two methods of cross-linking (CL) and non-crosslinking (NCL) probe-AuNPs hybridization were developed and compared.

**Results:**

The hybridization of positive targets with the two probe-AuNPs formed a polymeric network between the AuNPs which led to the aggregation of nanoparticles and color change from red to blue. Alternatively, in the NCL mode, the hybridization of complementary targets with the probe-AuNPs resulted in the increased electrostatic repulsion in nanoparticles and the increased stabilization against salt-induced aggregation. The CL and NCL assays had detection limits of 6.83 and 44.36 ng/reaction, respectively.

**Conclusion:**

The CL assay showed a higher sensitivity and specificity; in contrast, the NCL assay did not require optimizing and controlling of hybridization temperature and showed a higher response speed. However, both the developed methods are cost-effective and easy to perform and also could be implemented on-site or in local laboratories in low-resource countries.

**Supplementary Information:**

The online version contains supplementary material available at 10.1186/s12896-021-00691-w.

## Background

Bovine viral diarrhea virus (BVDV) belongs to the *Pestivirus* genus, *Flaviviridae* family. Previously, BVDV was divided into two species of BVDV-1 and BVDV-2 based on genetic and antigenic differences [1]. However, recently, International Committee on Taxonomy of Viruses (*ICTV*) has reorganized the *Pestivirus* genus to eleven species, including *Pestivirus* A-K. Under this new classification, BVDV-1 and BVDV-2 correspond to *Pestivirus* A and *Pestivirus* B, respectively. In the current study, we used previous names consistent with previous studies [2]. BVDV has a positive sense, the single-stranded RNA (ssRNA) genome, with an open reading frame (ORF) located between 5′ and 3′ untranslated regions (5’UTR, 3’UTR) [3]. BVDV causes diseases in ruminants, especially cattle [1]. Symptoms of BVDV infection in cattle include fever, diarrhea, abortion, stillbirth, congenital defective calf, decreased milk production, infertility, and even death. Infection of cows with BVDV during early pregnancy (the days 30–120) may lead to the birth of persistently infected (PI) calves. PI animals are significant because they remain viremic throughout their lives and serve as infection sources in cattle herds [4, 5]. BVDV is highly essential economically, as the mean annual loss directly attributed to BVDV is estimated to be 42.14€ per livestock [6]. The virus has spread in most countries, including Iran. Noaman and Nabinejad reported that the prevalence of BVDV in Isfahan Province, Iran, is 52.8 and 100% in cattle and farms, respectively [7]. In other provinces, the prevalence is almost the same [8, 9].

One way to control the disease and prevent it from spreading is to detect and cull infected animals, especially PI animals. Several methods have been used for BVDV detection including virus isolation (VI), immunohistochemistry, antigen capture enzyme-linked immunosorbent assay (AC-ELISA), reverse transcriptase polymerase chain reaction (RT-PCR), and real-time RT-PCR [1, 10]. VI is a “gold standard” diagnostic technique for BVDV (Sandvik, 2005); however, it is a labor-intensive and time-consuming process [10]. AC-ELISA has good sensitivity and specificity for detecting BVDV antigens; but, the presence of BVDV maternal antibodies or protein degradation influences results obtained by ELISA [1, 10]. Molecular techniques including RT-PCR and real-time RT-PCR have high accuracy; however, these assays are expensive and need specialized equipment and skilled personnel. Unfortunately, none of these assays can be performed in the field or in small local laboratories.

Using colorimetric biosensors for the rapid detection of biomolecules has recently attracted attentions [11]. Gold nanoparticles (AuNPs) are among the widely used nanomaterials in this field. AuNPs due to unique properties including easy synthesis, broad size and shape controllability, functionalization with biomolecules, long-term stability, and optical properties have been extensively applied to construct optical biosensors [11–13]. The surface plasmon resonance (SPR) is led to the solution of AuNPs with diameters of 5–30 nm showing a red color and the an absorption peak of around 520 nm; while, aggregated AuNPs’ color changes from red to purple or blue as SPR shifts to longer wavelengths [14, 15]. This phenomenon helps to design diagnostic tests based on color change in AuNPs, and thus, it is called AuNP-based colorimetric assay [16–18]. The assay has been applied for detecting of some pathogens including influenza virus [19, 20], *Klebsiella pneumonia* [12], *Brucella* spp. [21], *Salmonella* [22], *Escherichia coli* [23], and Middle East respiratory syndrome coronavirus [24].

**Fig. 1:**
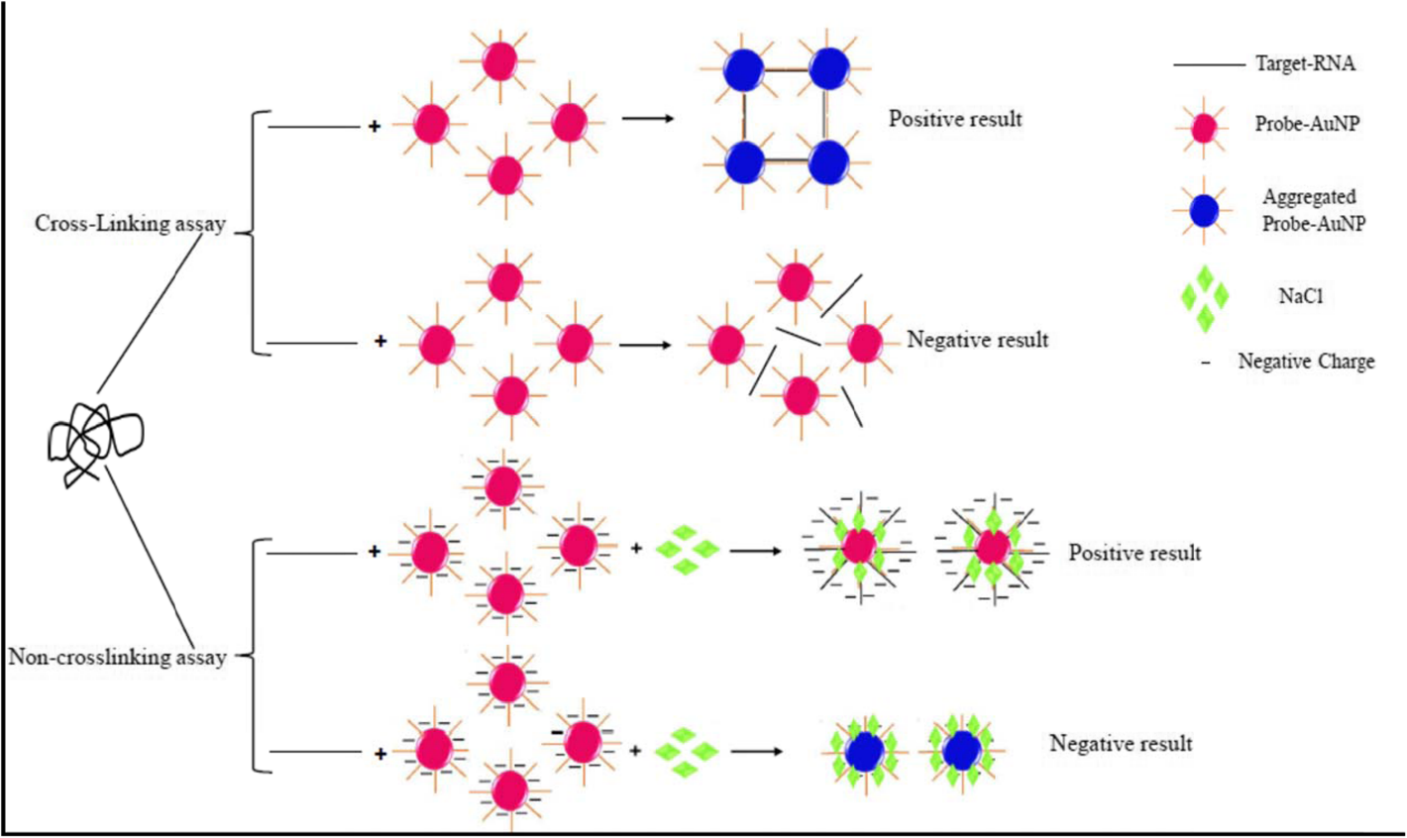
Schematic illustration of the colorimetric detection of nucleic acid target based on cross-linking and non-crosslinking probe-gold nanoparticle hybridization assays

AuNPs due to the negative charge of coated citrate anions have electrostatic repulsion and are stable in dispersed state. When salt is added, Na^+^ neutralizes the negative charge of citrate and thus, AuNPs are aggregated. However, the modification of AuNPs with thiolated DNA probes protects nanoparticles (NPs) against salt-induced aggregation due to the formation of a repulsive electric double layer by negative charges of citrate anions and DNA probes [25]. The thiol-linked oligonucleotide modified AuNPs (herein designated as ‘probe-AuNPs’) have a key application in nucleic acid detection by two platforms of cross-linking (CL) and non-crosslinking (NCL). In the CL method firstly developed by Mirkin et al., two contiguous and complementary probe-AuNPs to the target nucleic acid are used [26]. Through the hybridization of probe-AuNPs with the target sequence, a polymeric network is formed and causes color change in the solution from red to purple or blue; however, the color remains red in the absence of target nucleic acid (Fig. 1) [27, 28]. In latter mode, the assay is based on a non-crosslinking hybridization platform where one type of probe-AuNPs is used. In this assay, the aggregation of NPs is induced by increasing salt concentration. In the presence of the target sequence due to the hybridization of the target with the probe, NPs are protected from salt-induced aggregation and the solution remains red. However, in the presence of non-complementary or mismatched sequences, the solution’s changes from red to blue (Fig. 1) [23, 27, 29].

The use of probe-AuNPs for the colorimetric detection of nucleic acid targets represents an alternative to common amplification and non-amplification molecular assays. Herein, we developed and compared two BVDV-RNA detection methods using CL and NCL probe-AuNPs hybridization assays.

## Results

### Synthesis and characterization of AuNPs

**Fig. 2:**
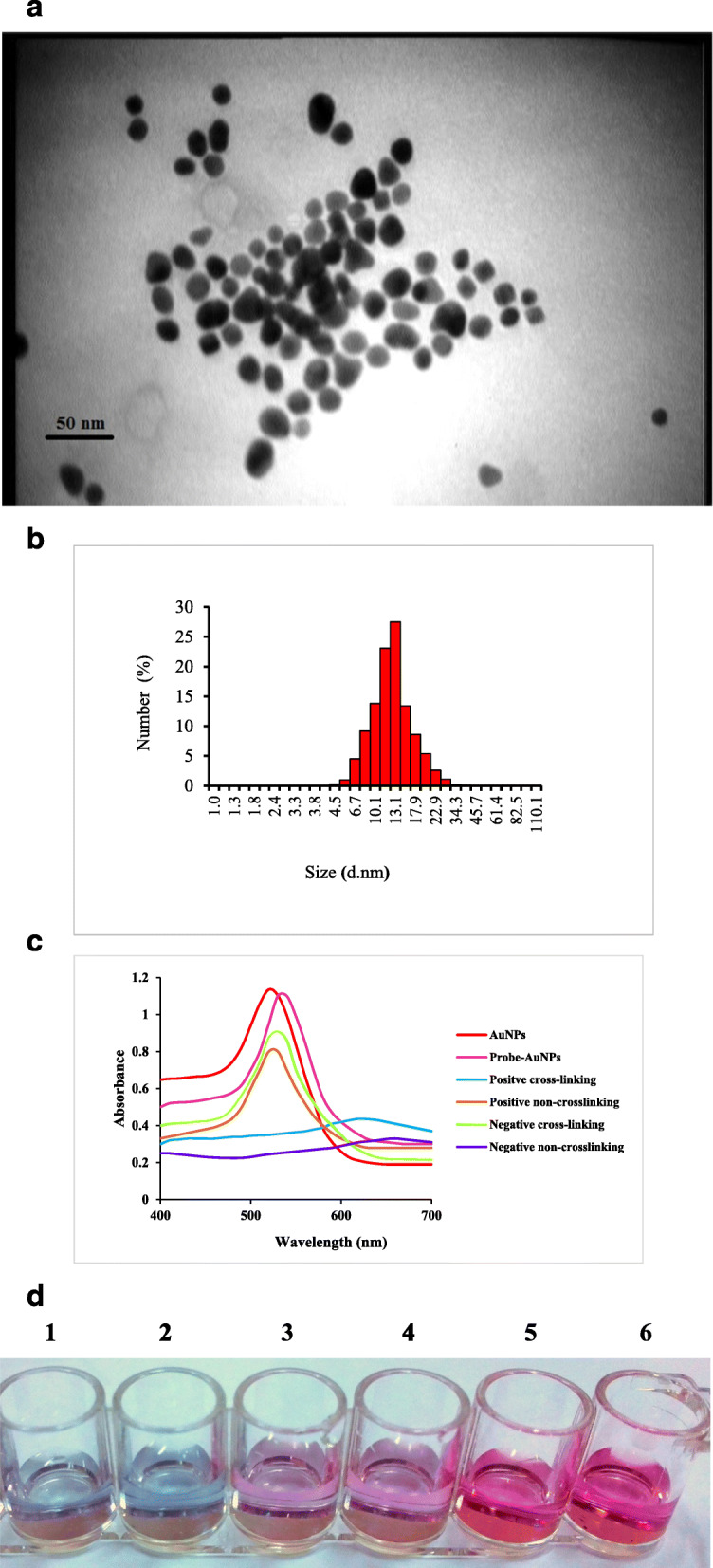
TEM image of AuNPs (**a**); AuNPs particle size distribution measured by DLS (**b**); UV-vis absorption spectra of the AuNPs, probe-AuNPs, positive and negative CL assays, and positive and negative NCL assays (**c**); Visual observation probe-AuNPs color changes in presence of positive and negative samples. From left to right, positive CL (well 1), negative NCL (well 2), negative CL (well 3), positive NCL (well 4), probe-AuNPs (well 5) and AuNPs (well 6)

Transmission electron microscopy (TEM) images showed that the AuNPs were synthesized and had a mean diameter of 13 nm (Fig. 2a). Dynamic light scattering (DLS) analysis revealed that more than 95% of the AuNPs were in the range of 7 to 22 nm (Fig. 2b). The UV-vis absorption spectrum of the free AuNPs exhibited a maximum peak at 520 nm (Fig. 2c and d).

### Functionalization of AuNPs

To modify the AuNPs, different probe to AuNP ratios including 50:1, 100:1, and 200:1 were used. When the probe concentration was low in the solution, the AuNPs were aggregated immediately after adding NaCl. However, the probe to AuNP ratio of 200:1, the probes protected the NPs from aggregation with NaCl. After functionalizing the AuNPs, the SPR peak shifted slightly from 520 nm to 525 nm (Fig. 2c and d). The probe-AuNPs were stable at the high concentration of NaCl (up to 2 M). However, the non-functionalized AuNPs were aggregated at the low concentration of NaCl (0.1 M), and the solution’s color changed from red to blue. The phosphates of the probe nucleotides increased the negative surface charge of the AuNPs, leading to the repulsion interaction of the AuNPs and the increased stability of the NPs [30].

### Optimization of CL and NCL probe-AuNPs hybridization reaction

To optimize the CL hybridization reaction, different denaturation temperatures of 80, 85, 90, and 95 °C at times of 1, 2, 3, 4, and 5 min and annealing temperatures of 42, 45, 50, and 55 °C were investigated. According to the best signal-to-noise level, temperatures of 90 °C for 3 min and 45 °C were applied for hybridization and annealing, respectively (Supplementary Table 1).

The optimization of NCL reaction was performed at different denaturation temperatures and times such as above, and finally, the temperature of 90 °C for 3 min was applied. Annealing was performed at room temperature for 30 min. After hybridization reaction reaction, the NaCl concentration was raised to 2 M (Supplementary Table 2).

### Analytical sensitivity of CL probe-AuNPs hybridization assay

**Fig. 3:**
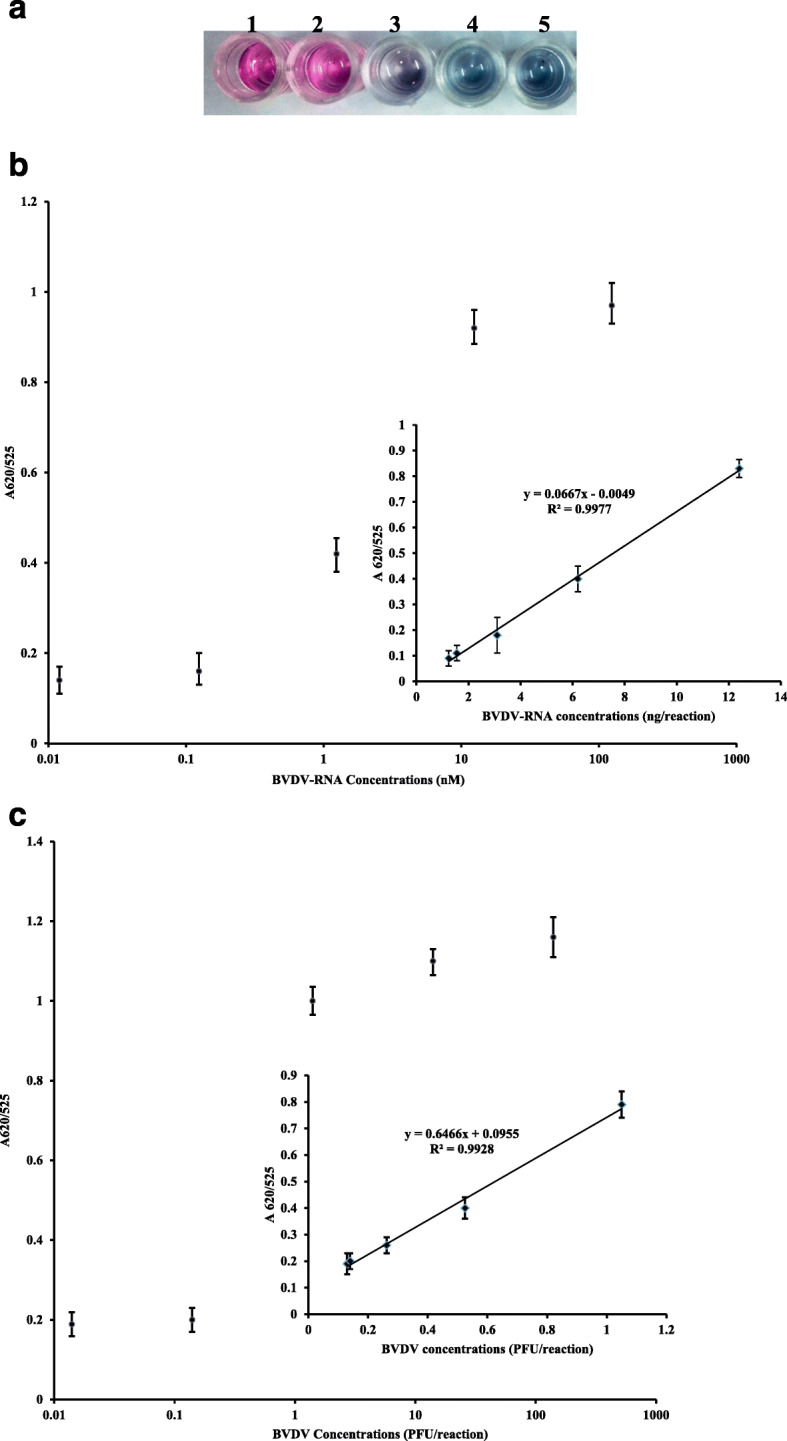
Visual color changes of developed CL probe-AuNPs hybridization assay at different BVDV-RNA concentrations. Wells 1–5 contain 0.012, 0.124, 1.24, 12.4, and 124 ng/reaction of RNA, respectively (**a**). Standard curve for the relationship between BVDV-RNA concentrations (ng/reaction) and absorbance ratio (A620/A525) (**b**) and BVDV concentrations (PFU/reaction) and absorbance ratio (A620/A525) (**c**). Inserts are the plot of linear correlation for RNA concentrations (1.24 to 12.4 ng/reaction) (**b**) and for BVDV concentrations (0.14 to 1.05 PFU/reaction) (**c**). The RNA concentrations 12.4 × 10^ (− 4) ˗ 12.4 × 10^ (− 6) showed absorbance ratio bellow 0.1

To evaluate the sensitivity of the developed methods, various BVDV-RNA concentrations of 12.4 × 10^ (− 6) to 124 ng/reaction were tested. The color change of the reaction was observed with the naked eye in the 10.4 ng/reaction of RNA (Fig. 3a). The limit of detection (LOD) was determined as 6.83 ng/reaction (Fig. 3b). Although the color change was not visually detectable at this RNA concentration, a change was observed in the wavelength using a spectrophotometer, indicating the high sensitivity of the newly developed method. The absorbance spectra of all the RNA concentrations were measured, and linear regression was determined. A linear relationship (*R* = 0.9977) was found between absorbance ratios at 620 nm/525 nm and various RNA concentrations in the range 1.24 to 12.4 ng/reaction (Fig. 3b). By increasing the RNA concentration, the solution’s color shifted from red to blue. The sensitivity of the developed method was determined according to plaque forming unite (PFU)/mL. The LOD was estimated to be 1.4 and 0.5 PFU/reaction, respectively, with the naked eye and a spectrophotometer (Fig. 3c). A linear regression (*R* = 0.9928) was found between absorbance ratios and number of viruses in the range 0.14 to 1.05 PFU/reaction (Fig. 3c).

The sensitivity of RT-nested multiplex-PCR and real-time RT-PCR were obtained to be 12.4 × 10^ (− 4) and 12.4 × 10^ (− 5) ng/reaction, respectively, and thus, they were both more sensitive than the probe-AuNPs hybridization assay.

### Analytical sensitivity of NCL probe-AuNPs hybridization assay

**Fig. 4:**
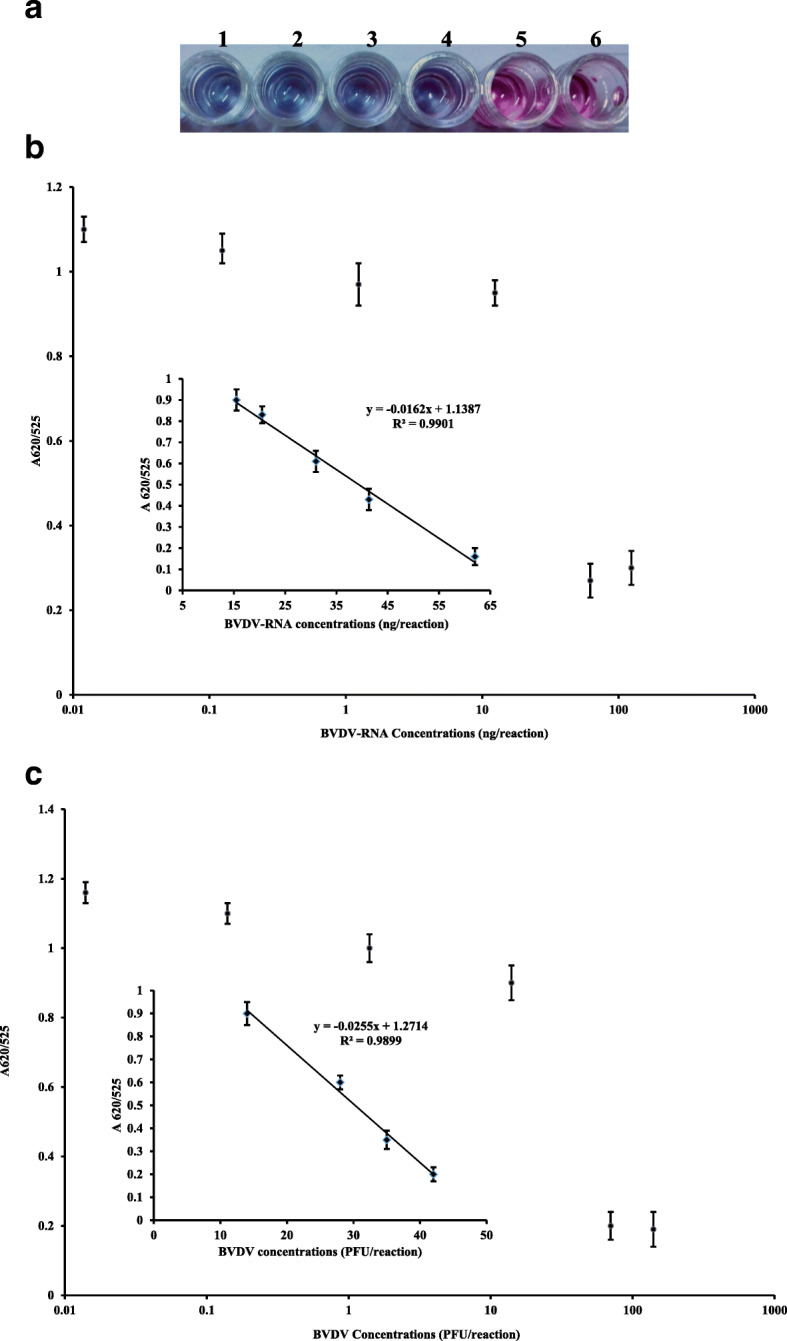
Visual color changes of developed NCL probe-AuNPs hybridization assay at different BVDV-RNA concentrations. Wells 1–6 contain 0.012, 0.124, 1.24, 12.4, 62, and 124 ng/reaction of RNA, respectively (**a**). Standard curve for the relationship between BVDV-RNA concentrations (ng/reaction) and absorbance ratio (A620/A525) (**b**) and BVDV concentrations (PFU/reaction) and absorbance ratio (A620/A525) (**c**). Inserts are the plot of linear correlation for RNA concentrations (15.5 to 62 ng/reaction) (**b**) and for BVDV concentrations (14 to 42 PFU/reaction) (**c**). The RNA concentrations 12.4 × 10^ (− 4) ˗12.4 × 10^ (− 6) showed absorbance ratio bellow 0.1

The sensitivity of the NCL assay was found 62 and 44.36 ng/reaction of RNA with the naked eye and a spectrophotometer, respectively. The sensitivity of the NCL method according to PFU was calculated 42 and 10.64 PFU/mL using visual and instrument, respectively. Figure 4 shows the linear relationship between the absorption A620/A525 ratio and the increased BVDV-RNA concentration in the range of 15.5 to 62 ng/reaction (R2 = 0.9901). In contrast to the CL assay, in the NCL method by increasing the RNA concentration, the solution’s color shifted from blue to red.

### Analytical specificity of CL and NCL probe-AuNPs hybridization assays

Different sequences of the 5′ untranslated region (5′UTR) of BVDV-RNA were aligned and a conserved region of the viral genome was selected as the target. Two probes were designed to detect and hybridize with the target sequence. The specificity of the probes was assessed by basic local alignment search tool (BLAST) search, and results showed that the probes were specific for BVDV-1 and BVDV-2.

**Fig. 5:**
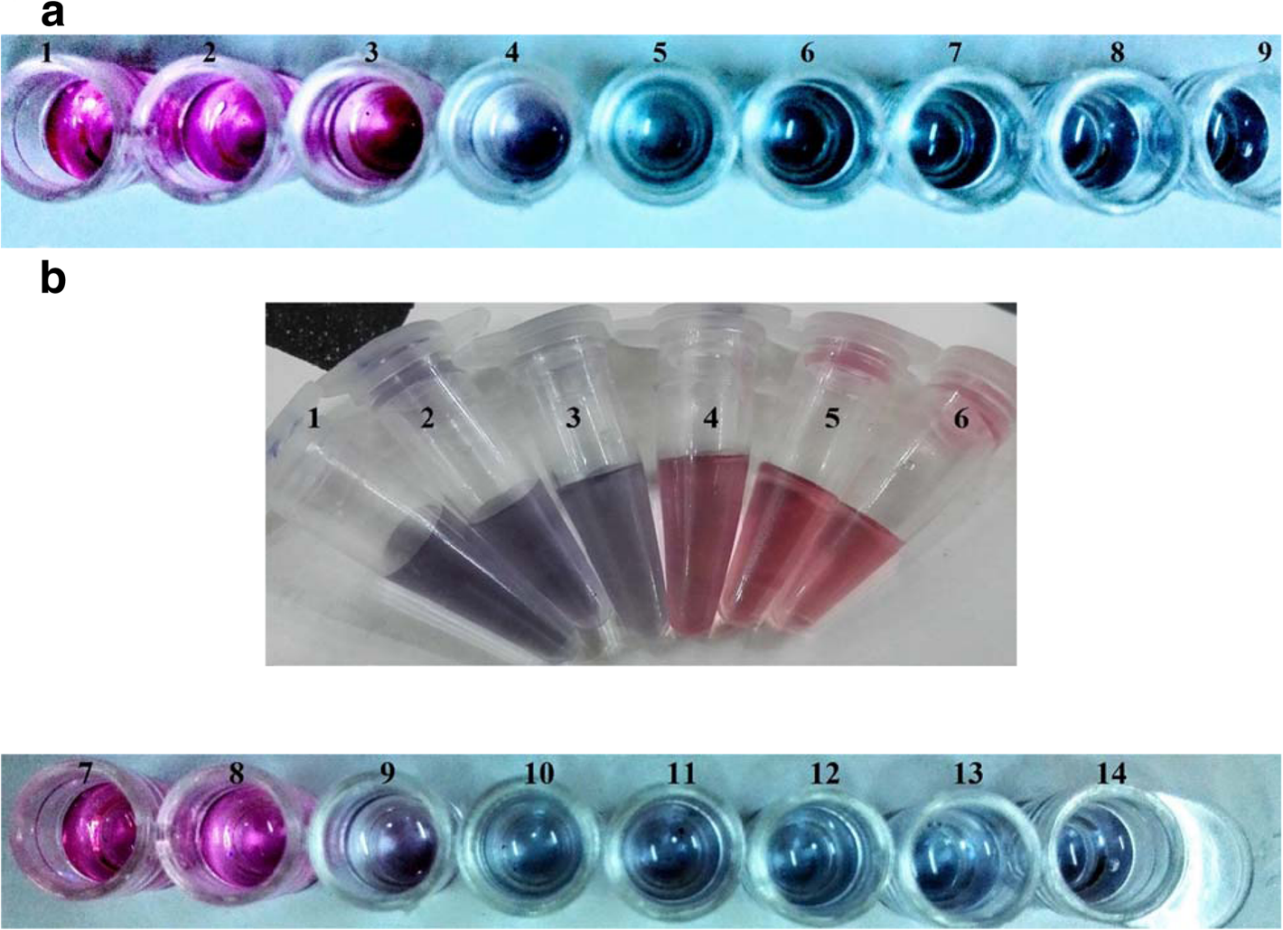
Visual observation of CL and NCL probe-AuNPs hybridization assays in presence of negative and positive specimens. CL probe-AuNPs hybridization assay; from left to right, 1 mismatch target; 2 deleted target; 3 negative field sample; 4 BVDV-NADL; 5 BVDV-2; 6 DNA target; 7–9 positive field sample; **a**. NCL probe-AuNPs hybridization assay; from left to right, 1 mismatch target; 2 deleted target; 3 MDBK cell line; 4 BVDV-NADL 5 BVDV-2; 6 DNA target; 7–8 positive field samples; 9–14 negative field samples (**b**)

The new assays were performed on several positive and negative control samples. All the negative controls including unmatched and mismatch DNA sequences, uninfected Madin–Darby bovine kidney (MDBK) and Razi Bovine Kidney (RBK) cell lines, blood samples of healthy bovines, foot-and-mouth disease virus (FMDV), bovine leukemia virus cultivated on fetal lamb kidney (BLV-FLK), bovine-herpesvirus 1 (BoHV-1), *Mycobacterium bovis*, *E. coli* O157:H7, and *Pasteurella multocida* were negative for both the newly developed assays. The reactions’ color remained red in the CL assay, while the solutions’ color changed from red to blue in the NCL assay. However, for the CL assay, positive controls including references and archival samples changed the reaction’s color from red to blue and were considered positive (Fig. 5a). In the NCL method, in contrast, the color of all the positive samples remained red (Fig. 5b).

### Relative diagnostic sensitivity and specificity

Fifty clinical samples suspected of infection with BVDV were investigated simultaneously using RT-nested multiplex-PCR, real-time RT-PCR, and CL and NCL probe-AuNPs hybridization assays. The relative diagnostic sensitivity and specificity of the CL and NCL developed methods were calculated according to the following formula: sensitivity: $$ \raisebox{1ex}{$ TP$}\!\left/ \!\raisebox{-1ex}{$ TP+ FN$}\right. $$ and specificity: $$ \raisebox{1ex}{$ TN$}\!\left/ \!\raisebox{-1ex}{$ TN+ FP$}\right. $$, where TP, FN, TN, and FP are true positive, false negative, true negative, and false positive, respectively. Of the 50 specimens, 20 were detected as BVDV using RT-nested multiplex-PCR and real-time RT-PCR; however, the CL and NCL assays could detect 18 and 17 of the specimens, respectively. Samples unidentified by the new methods were confirmed as BVDV by culturing the samples on cells, and thus, were considered true positive. No samples were detected positive by the CL probe-AuNPs hybridization assay and negative by the other two methods. However, one true negative sample was detected as BVDV in the NCL assay (Supplementary Table 3). Based on these results, the relative diagnostic sensitivity and specificity of the newly developed CL method were obtained 90 and 100%, respectively. The overall agreement was 96% between the molecular tests and the CL assay. The diagnostic sensitivity and specificity of the NCL assay were found 85 and 97%, respectively. An overall agreement of 92% was found between the molecular tests and the NCL method. The two developed assays had an overall agreement of 96%.

## Discussion

The real-time detection of pathogenic agents has a high interest in various fields including medicine, veterinary science, biosecurity, bioterrorism, and environmental science [31]. The AuNPs colorimetric based assays have the capacity to be automated as chips. By designing and using specific probes, a wide range of pathogenic microorganisms, including bacteria, viruses and fungi can be identified simultaneously [32]. Citrate coated AuNPs prepared by the citrate reduction method are highly favorable for biological assays because of easy synthesis and high yield. Citrate as a capping agent on the surface of AuNPs can be easily replaced by other capping agents such as thiol that help binding of biological compounds to AuNPs [33, 34].

In the current study, we developed two methods of CL and NCL probe-AuNPs hybridization assays to propose novel, fast, sensitive, and enzyme-free methods for the detection of both BVDV-1 and BVDV-2. The first report about using nanomaterials for the detection of viruses was related to 1997, in which AuNPs coupled with silver staining were applied to detect of human papillomavirus [13, 35]. Since then, NPs, especially AuNPs, have been used to identify viruses in several cases. The basis of these methods is usually the detection of proteins, antigens, or nucleic acid of the virus. Due to the specificity of BVDV-RNA sequence, the proposed methods were designed based on virus nucleic acid detection. The most conserved region of the BVDV-RNA is the 5′ UTR sequence, which was detected by both the developed methods. Assessing the specificity of the designed methods showed that this sequence was very suitable for BVDV identification.

Visual and instrument color change detections showed that the CL probe-AuNPs hybridization assay was more sensitive than NCL method. Visual and instrument LOD for CL assay was 10.24 and 6.83 ng/reaction. Moreover, the LOD for the NCL assay with the naked eye and a spectrophotometer was estimated to be 62 and 44.36 ng/reaction, respectively. However, the both methods exhibited lower sensitivity than RT-nested multiplex PCR and real-time RT-PCR. The reason is that the developed methods are not based on target amplification. The NCL method requires a greater number of target RNA to prevent the salt-induced aggregation; therefore, this method is less sensitive than the CL assay. Previously, our research group used unmodified AuNPs assay to detect unamplified BVDV-RNA; however, the two recently developed methods are more sensitive than unmodified AuNPs assay because free probes may be adsorbed to AuNPs and reduce the sensitivity of unmodified AuNPs assay [36]. When peptide nucleic acid (PNA)-probe was used instead of DNA-probe in unmodified AuNPs assay, the assay’s sensitivity increased and was almost equivalent to that of the CL assay [37].

The results of analytical specificity showed that the developed CL and NCL assays were selective for the detection of BVDV-RNA. However, the intensity of color difference between positive and negative control samples was greater in the CL assay than in the NCL method, and thus, detection was easier in the CL assay. The results of relative diagnostic specificity showed that the CL method was more specific than the NCL assay. The probes were designed in a tail-to-tail manner and each probe had a 15 bp specific sequence complemented to adjacent sequences in the target RNA. Therefore, it is feasible to identify a continuous 30 bp region on the genome. This mode action enhances the assay’s specificity and decreases the nonspecific binding of probes to the target genome [32]. In the NCL method, only one probe was used and, probably for this reason, specificity was lower in this method than in the CL assay. Compared to RT-nested multiplex-PCR and real-time RT-PCR, the CL assay had the same specificity but NCL had lower specificity. One of the real negative samples was detected as positive in NCL, which may be due to the nonspecific binding of nucleic acids to the probe-AuNPs. When the salt concentration increases, the negative charge of nucleic acids is neutralized to some extent, causing to increase non-specific bindings.

The comparison of the developed CL and NCL assays showed that the both methods were simple, did not need trained personnel or expensive instruments, did not require enzyme or target amplification, and could be read with the naked eye; however, the CL method was more sensitive and specific. The NCL assay required more target RNA than the CL method to identify positive samples. Sato et al reported that a single DNA target was sufficient to cross-link two AuNPs together whereas the NCL assay required about 200 target molecules per one AuNP [38]. The advantages of the NCL method included using one type nano-probe, not requiring the optimization and control of hybridization temperature, and having higher reaction speed. Compared to molecular methods investigated in the current study, although the CL and NCL assays were less sensitive and specific, they were faster and did no need reverse transcription. The developed assays can be directly performed on total RNA and do not require the conversion of RNA to cDNA or amplification.

The design of probes is highly critical in the CL and NCL assays. The both probes, in addition to having specific complementary sequences to BVDV-RNA, had a 10 base chain of adenine nucleotides at the end, which was connected to AuNP. Poly(A) tails acted as a spacer and reduced the steric hindrance between specific sequences of probes and the AuNP surface, thereby significantly improving the hybridization efficiency [39, 40].

The developed methods cannot detect whether the virus is active or inactive (live or dead). This limitation also exits in other molecular methods, as they can only detect the presence of the virus. The newly developed CL and NCL assays may not be able to find BVDV during the early or end stage of the disease when the virus titer is low. However, these novel methods can effectively identify PI cows or BVDV in the acute phase of the disease and can be performed in the field.

## Conclusion

We developed two methods of CL and NCL colorimetric AuNPs to detect BVDV that were cost-effective, easy to perform, robust, and rapid and also could be performed on-site or in local laboratories in low-resource countries. Comparison of the two developed methods showed that the CL assay had higher sensitivity and specificity whereas the NCL assay had a higher response speed. These novel methods are beneficial for enhancing BVDV diagnostic efficiency, especially detecting PI animals, effectively controlling the spread of the disease, and reducing losses in cattle farms.

## Materials and methods

### Reference isolates, clinical isolates, and other pathogens

The reference isolates of NADL-BVDV (BVDV-1) and BVDV-2 (Razi strain) were purchased from the Razi Vaccine and Serum Research Center (Hessarak, Iran) and used as positive controls. The viruses were inoculated to the MDBK cell line in RPMI1640 fed with 10% FBS (Gibco, Scotland) and incubated at 37 °C under 5% CO_2_ to form cytopathic effects (CPEs). The titers of the viruses were measured by the PFU assay and calculation of 50% tissue culture infective dose (TCID_50_) using the Reed and Munech method. The reference strains were used to develop and optimize the hybridization reaction conditions.

Clinical samples were taken from fifty calves with clinical signs of BVDV including diarrhea, fever, respiratory disorders, and still birth. Buffy coats of blood samples were subjected to RNA extraction.

Negative controls included several bacterial and viral bovine pathogens, blood samples of two healthy bovines, and the MDBK cell line. Bacterial pathogens included *M. bovis* (BCG), *E. coli* O157:H7 (ATCC 35218), and *P. multocida* (ATCC 15742). The used viral pathogens were BLV-FLK, FMDV type O IRAN/1/2010, and BoHV-1.

### Ethics approval

The study was approved by Ethics Committee of Shahid Chamran University of Ahvaz (No:92.9.24/scu.ac.ir). Written consent from the farms owners were obtained before the study. Blood specimens were consisted of field samples collected during clinical examination of animals. According to animal ethics committee, anesthesia is not required to blood sampling from calves; however, Sampling was performed by experienced veterinarians to minimize animal suffering and pain. The results of the tests were communicated to the farm owners. According to the recommendation of the National Veterinary Organization, farmers were advised to cull BVDV-infected animals and send them to the slaughterhouse or if they want to keep livestock, the infected cases should be quarantined and receive supportive treatment. The ethics approval and informed consent were not required for the cell lines used in this study.

### RNA and DNA extraction

RNA was extracted from buffy coats of the blood samples, reference and archival strains, uninfected MDBK and RBK cell lines, and FMDV using the RNA extraction kit (Bioneer; South Korea) following the manufacturer’s instructions.

DNA was extracted from *M. bovis*, *E. coli* O157:H7, *P. multocida*, BLV-FLK, and BoHV-1 using the DNA extraction kit (Bioneer; South Korea) following the manufacturer’s instructions. All the extracted nucleic acids were stored at − 80 °C until used.

### RT-nested multiplex-PCR and real-time RT-PCR

The RNA samples were reversely transcripted to cDNA using the TaKaRa cDNA synthesis kit (Japan) according to the manufacturer’s instructions. Nested multiplex-PCR was performed to detect BVDV-1 and BVDV-2 following Gilbert’s et al. method [41]. The target location of the primers was the NS5B of BVDV-RNA. These primers recognized the fragments of the 369 bp length of BVDV-1 or the 615 bp of BVDV-2 (Table 1). The real-time RT-PCR was performed using Applied Biosystems (USA) as described by Aebischer et al (Table 1) [42].

**Table 1:** Probes, primers, and target sequences used in CL and NCL probe-AuNP hybridization assay, RT-nested multiplexPCR, and real-time RT-PCR

Assay	Name	Sequence (5′ 3′)	Reference
CL or NCL probe-AuNP hybridization assay	Probe-A	TTCAGCCATCCAACG A(10) SH	This paper
	Probe-B	SHA(10) TACCCTGTACTCAGG	This paper
	Target	CGTTGGATGGCTGAAGCCCTGAGTACAGGGTA	This paper
	Mismatch target	CGATGGATCGCTGAAGCCCTGAGTACGGGCTA	This paper
	Deleted target	CGTGATGGCTGAAGCCCTGAGTACGGTA	This paper
RT-nested multiplexPCR	ExF	AAGATCCACCCTTATGA(A/G)GC	[41]
	ExR	AAGAAGCCATCATC(A/C)CCACA	[41]
	InF BVDV-1	TGGAGATCTTTCACACAATAGC	[41]
	Inf BVDV-2	GGGAACCTAAGAACTAAATC	[41]
	InR	GCTGTTTCACCCAGTT(A/G)TACAT	[41]
Real-time RT-PCR	Pesti-3F	CCTGAGTACAGGRTAGTCGTCA	[42]
	Pesti-4R	GGCCTCTGCAGCACCCTATCA	[42]
	TQ-Pesti-Probe	FAM-TGCYAYGTGGACGAGGGCATGC-BHQ-1	[42]

### Synthesis of AuNPs

AuNPs were prepared by the citrate reduction of tetrachloroauric acid (HAuCl_4_). Briefly, 50 mL of 0.01% HAuCl_4_ was boiled. Then, 0.6 mL of 1% sodium citrate solution was added quickly after boiling stopped and stirring continued. After a few minutes, the solution’s color turned from yellow to gray, blue, purple, and finally red. Stirring continued to cool the solution to room temperature.

### Characterization of AuNPs

TEM (Zeiss Em10c, 80 kv, Germany) and DLS with a Malvern instrument (UK) model Nano zs (red badge) ZEN 3600 were used for assessing AuNPs. UV–vis absorption spectra were recorded using UV–visible spectrophotometry (GBC, Cintra 101, Australia).

### Design of probes

The 5′UTR of the BVDV-RNA is the most conserved region of the genome; therefore, this sequence was used to design probes. Thirty BVDV reference sequences (24 BVDV-1 and six BVDV-2 strains) were randomly retrieved from the GenBank database and aligned using ClustalX. The most conserved regions were selected and assessed using the BLAST to select BVDV specific sequences. The length of each probe was considered 15 nucleotides. Complementary sequences at the ends of the probes were avoided. The melting temperatures of the probes were considered within a narrow range. For a better conjugation of the probes to AuNPs, 10 bases of A and the thiol group were added to the connecting end of the probes to AuNPs (Table 1).

### Functionalization of AuNPs

Capping AuNPs with 5′-thiol terminated probe (probe A) and 3′-thiol terminated probe (probe B) was performed using the [18]. For this purpose, 15 μL of each probe (10 μM) was added separately to 35 μL of AuNPs (20 nM). Subsequently, the solutions were incubated at room temperature for 16 h. The conjugation solutions were then brought to the final concentration of 0.1 M NaCl and 10 mM phosphate buffer by adding 20 μL of connection buffer containing phosphate buffer/NaCl (35 mM/350 mM) within 16 h. Then, the solution was centrifuged three times at 12000 rpm for 5 min. After each centrifugation, the supernatant was removed and the oily pellet was resuspended in assay buffer (10 mM phosphate buffer/0.3 M NaCl). The final concentration of the functionalized AuNPs was considered 10 nM. This method was used for the two designed probes. The two probes (A and B) were used in the CL assay; while, one of them (probe A or B) was used in the NCL assay.

### Hybridization reaction of CL probe-AuNPs colorimetric assay

The hybridization of probe-AuNPs and the target was performed by adding 10 μL of probe-AuNP (the final concentration of each probe was approximately 0.85 μM) to 5 μL of the target. The mixture was denatured at 95 °C for 3 min and hybridized at 45 °C for 10 min. Color changes were assessed with the naked eye and a UV-visible spectrophotometer.

### Hybridization reaction of NCL probe-AuNPs colorimetric assay

The NCL reaction mixture of 20 μL volume contained 5 μL of target, 4 μL of phosphate buffer (100 mM), 10 μL of probe-AuNPs (A or B), and water. The mixture was heated at 95 °C for 3 min; then, incubated at room temperature for 30 min. Finally, 20 μL of NaCl (4 M) was added to the reaction. Color changes was assessed by naked eye and UV-visible spectrophotometer.

### Analytical sensitivity and specificity of CL and NCL probe-AuNPs hybridization assays

The sensitivity of the developed methods was assayed by calculating limit of detection (LOD) according to the following formula: LOD = 3 standard deviation of the blank. RNA was extracted from the supernatant of BVDV-infected cells. The amount of extracted RNA was measured using a NanoDrop (*Thermo* Scientific; USA) and was equal to 248 ng/μL. The titer of virus was calculated 28 PFU/μL. Then, ten-fold serial dilutions of RNA extracted from the supernatant of BVDV-infected cells (12.4 × 10^(− 6) to 124 ng/reaction) were prepared and analyzed using CL and NCL probe-AuNPs hybridization assays. Moreover, the sensitivity of the new methods was analyzed according to ten-fold serial dilutions of the virus (0.014 to 140 PFU/reaction). The same templates were analyzed by RT-nested multiplex-PCR and real-time RT-PCR to compare the sensitivity of these methods with the newly designed assays.

To determine the reaction specificity, several viral and bacterial bovine pathogens (indicated above), bloods of healthy bovines, two different cell lines, and reference and archival strains were assayed by CL and NCL probe-AuNPs hybridization reactions.

### Relative diagnostic sensitivity and specificity

A total of 50 suspected clinical samples were analyzed in parallel using the developed methods, RT-nested multiplex-PCR, and real-time RT-PCR as descripted above.

## Supplementary Information

**Additional file 1: Supplementary Table 1**. The optimization of cross-linking probe-AuNPs hybridization reaction. **Supplementary Table 2**. The optimization of non-crosslinking probe-AuNPs hybridization reaction. **Supplementary Table 3**. Results of clinical samples analysis using RT-nested multiplex-PCR, real-time RT-PCR, and CL and NCL probe-AuNPs hybridization assays.

## Data Availability

The datasets used and/or analyzed during the current study are available from the corresponding author on reasonable request.

## Abbreviations

BVDV: Bovine viral diarrhea virus
PI: Persistent infection
AuNPs: Gold nanoparticles
CL: Cross-linking
NCL: Non-crosslinking
*ICTV*: International Committee on Taxonomy of Viruses
ssRNA: Single-stranded RNA
ORF: Open reading frame
5’UTR, 3’UTR: 5′ and 3′ untranslated regions
VI: Virus isolation
AC-ELISA: Antigen capture enzyme-linked immunosorbent assay
RT-PCR: Reverse transcriptase polymerase chain reaction
SPR: Surface plasmon resonance
NPs: Nanoparticles
MDBK: Madin–Darby bovine kidney
CPEs: Cytopathic effects
PFU: Plaque forming unit
TCID_50_: 50% tissue culture infective dose
RBK: Razi Bovine Kidney
BLAST: Basic local alignment search tool
TEM: Transmission Electron Microscopy
DLS: Dynamic light scattering
HAuCl_4_: Tetrachloroauric acid
BLV-FLK: Fetal lamb kidney
FMDV: Foot-and-mouth disease virus
BoHV-1: Bovine-herpesvirus 1

## References

[1] Spetter MJ, Louge Uriarte EL, Armendano JI, Morrell EL, Cantón GJ, Verna AE, Dorsch MA, Pereyra SB, Odeón AC, Saliki JT, González Altamiranda EA (2020). Detection methods and characterization of bovine viral diarrhea virus in aborted fetuses and neonatal calves over a 22-year period. Braz J Microbiol.

[2] Walz PH, Chamorro MF, MF S, Passler T, van der Meer F, RW A (2020). Bovine viral diarrhea virus: an updated American College of Veterinary Internal Medicine consensus statement with focus on virus biology, hosts, immunosuppression, and vaccination. J Vet Intern Med.

[3] Liang H, Geng J, Bai S, Aimuguri A, Gong Z, Feng R, Shen X, Wei S (2019). TaqMan real-time PCR for detecting bovine viral diarrhea virus. Pol J Vet Sci.

[4] Kaveh A, Merat E, Samani S, Danandeh S, Soltan NS (2017). Infectious causes of bovine abortion in Qazvin Province, Iran. Arch Razi Inst.

[5] Garoussi MT, Mehrzad J, Nejati A (2019). Investigation of persistent infection of bovine viral diarrhea virus (BVDV) in Holstein dairy cows. Trop Anim Health Prod.

[6] Pinior B, Garcia S, Minviel JJ, Raboisson D (2019). Epidemiological factors and mitigation measures influencing production losses in cattle due to bovine viral diarrhoea virus infection: a meta-analysis. Transbound Emerg Dis.

[7] Noaman V, Nabinejad AR. Seroprevalence and risk factors assessment of the three main infectious agents associated with abortion in dairy cattle in Isfahan province, Iran. Trop Anim Health Prod. 2020:1–9. 10.1007/s11250-020-02207-8 31983025

[8] Ghaemmaghami S, Ahmadi M, Deniko A, Mokhberosafa L, Bakhshesh M (2013). Serological study of BVDV and BHV-1 infections in industrial dairy herds of Arak, Iran. Iran J Vet Sci Technol.

[9] Erfani AM, Bakhshesh M, Fallah MH, Hashemi M (2019). Seroprevalence and risk factors associated with bovine viral diarrhea virus and bovine herpes virus-1 in Zanjan Province, Iran. Trop Anim Health Prod.

[10] Khodakaram-Tafti A, Farjanikish G (2017). Persistent bovine viral diarrhea virus (BVDV) infection in cattle herds. Iran J Vet Res.

[11] Jin R, Zhai L, Zhu Q, Feng J, Pan X. Naked-eyes detection of largemouth bass ranavirus in clinical fish samples using gold nanoparticles as colorimetric sensor. Aquaculture. 2020;735554.

[12] Ahmadi S, Kamaladini H, Haddadi F, Sharifmoghadam MR (2018). Thiol-capped gold nanoparticle biosensors for rapid and sensitive visual colorimetric detection of *Klebsiella pneumoniae*. J Fluoresc.

[13] Draz MS, Shafiee H (2018). Applications of gold nanoparticles in virus detection. Theranostics.

[14] Naderi M, Hosseini M, Ganjali MR (2018). Naked-eye detection of potassium ions in a novel gold nanoparticle aggregation-based aptasensor. Spectrochim Acta A Mol Biomol Spectrosc.

[15] Swierczewska M, Liu G, Lee S, Chen X (2012). High-sensitivity nanosensors for biomarker detection. Chem Soc Rev.

[16] Wang G, Akiyama Y, Shiraishi S, Kanayama N, Takarada T, Maeda M (2017). Cross-linking versus non-cross-linking aggregation of gold nanoparticles induced by DNA hybridization: a comparison of the rapidity of solution color change. Bioconjug Chem.

[17] Conde J, de la Fuente JM, Baptista PV (2010). RNA quantification using gold nanoprobes-application to cancer diagnostics. J Nanobiotechnol.

[18] Baptista P, Doria G, Henriques D, Pereira E, Franco R (2005). Colorimetric detection of eukaryotic gene expression with DNA-derivatized gold nanoparticles. J Biotechnol.

[19] Liu Y, Zhang L, Wei W, Zhao H, Zhou Z, Zhang Y, Liu S (2015). Colorimetric detection of influenza a virus using antibody-functionalized gold nanoparticles. Analyst.

[20] Lee C, Gaston MA, Weiss AA, Zhang P (2013). Colorimetric viral detection based on sialic acid stabilized goldnanoparticles. Biosens Bioelectron.

[21] Sattarahmady N, Tondro G, Gholchin M, Heli H (2015). Gold nanoparticles biosensor of Brucella spp. genomic DNA: visual and spectrophotometric detections. Biochem Eng J.

[22] Khan SA, Singh AK, Senapati D, Fan Z, Ray PC (2011). Targeted highly sensitive detection of multi-drug resistant salmonella DT104 using gold nanoparticles. Chem Commun.

[23] Bakthavathsalam P, Rajendran VK, Mohammed JAB (2012). A direct detection of Escherichia coli genomic DNA using gold nanoprobes. J Nanobiotechnol.

[24] Kim H, Park M, Hwang J, Kim JH, Chung D-R, Lee K-s (2019). Development of label-free colorimetric assay for MERS-CoV using gold nanoparticles. ACS Sensors.

[25] Zhao W, Brook MA, Li Y (2008). Design of gold nanoparticle-based colorimetric biosensing assays. ChemBioChem.

[26] Mirkin CA, Letsinger RL, Mucic RC, Storhoff JJ (1996). A DNA-based method for rationally assembling nanoparticles into macroscopic materials. Nature.

[27] Ahmadpour-Yazdi H, Hormozi-Nezhad M, Abadi A, Sanati MH, Kazemi B (2013). Colorimetric assay for exon 7 SMN1/SMN2 single nucleotide polymorphism using gold nanoprobes. Bioimpacts.

[28] Soo P-C, Horng Y-T, Chang K-C, Wang J-Y, Hsueh P-R, Chuang C-Y, Lu CC, Lai HC (2009). A simple gold nanoparticle probes assay for identification of *Mycobacterium tuberculosis* and Mycobacterium tuberculosis complex from clinical specimens. Mol Cell Probes.

[29] Mollasalehi H, Yazdanparast R (2012). Non-crosslinking gold nanoprobes for detection of nucleic acid sequence-based amplification products. Anal Biochem.

[30] Esmaeili-Bandboni A, Amini SM, Faridi-Majidi R, Bagheri J, Mohammadnejad J, Sadroddiny E (2018). Cross-linking gold nanoparticles aggregation method based on localised surface plasmon resonance for quantitative detection of miR-155. IET Nanobiotechnol.

[31] Zhang W, Guo S, Carvalho WSP, Jiang Y, Serpe MJ (2016). Portable point-of-care diagnostic devices. Anal Methods.

[32] Vaseghi A, Safaie N, Bakhshinejad B, Mohsenifar A, Sadeghizadeh M (2013). Detection of *Pseudomonas syringae* pathovars by thiol-linked DNA–gold nanoparticle probes. Sensors Actuators B Chem.

[33] Baptista P, Pereira E, Eaton P, Doria G, Miranda A, Gomes I, Quaresma P, Franco R (2008). Gold nanoparticles for the development of clinical diagnosis methods. Anal Bioanal Chem.

[34] Kimling J, Maier M, Okenve B, Kotaidis V, Ballot H, Plech A (2006). Turkevich method for gold nanoparticle synthesis revisited. J Phys Chem B.

[35] Zehbe I, Hacker GW, Su H, Hauser-Kronberger C, Hainfeld JF, Tubbs R (1997). Sensitive in situ hybridization with catalyzed reporter deposition, streptavidin-Nanogold, and silver acetate autometallography: detection of single-copy human papillomavirus. Am J Pathol.

[36] Heidari Z, Rezatofighi SE, Rastegarzadeh S (2016). A novel unmodified gold nanoparticles-based assay for direct detection of unamplified bovine viral diarrhea virus-RNA. J Nanosci Nanotechnol.

[37] Askaravi M, Rezatofighi SE, Rastegarzadeh S, Shapouri MRSA (2017). Development of a new method based on unmodified gold nanoparticles and peptide nucleic acids for detecting bovine viral diarrhea virus-RNA. AMB Express.

[38] Sato K, Hosokawa K, Maeda M (2003). Rapid aggregation of gold nanoparticles induced by non-cross-linking DNA hybridization. J Am Chem Soc.

[39] He Z, Yang H (2018). Colourimetric detection of swine-specific DNA for halal authentication using gold nanoparticles. Food Control.

[40] Demers LM, Mirkin CA, Mucic RC, Reynolds RA, Letsinger RL, Elghanian R, Viswanadham G (2000). A fluorescence-based method for determining the surface coverage and hybridization efficiency of thiol-capped oligonucleotides bound to gold thin films and nanoparticles. Anal Chem.

[41] Gilbert S, Burton K, Prins S, Deregt D (1999). Typing of bovine viral diarrhea viruses directly from blood of persistently infected cattle by multiplex PCR. J Clin Microbiol.

[42] Aebischer A, Wernike K, Hoffmann B, Beer M (2014). Rapid genome detection of Schmallenberg virus and bovine viral diarrhea virus by use of isothermal amplification methods and high-speed real-time reverse transcriptase PCR. J Clin Microbiol.

