# Supplementary material for: Development and comparison of cross-linking and non-crosslinking probe-gold nanoparticle hybridization assays for direct detection of unamplified bovine viral diarrhea virus-RNA
Source: BMC Biotechnol. 2021 Apr 23;21:30. doi: 10.1186/s12896-021-00691-w (PMC8063192; doi:10.1186/s12896-021-00691-w)
Supplement: Supplementary file 1 — Additional file 1: Supplementary Table 1. The optimization of cross-linking probe-AuNPs hybridization reaction. Supplementary Table 2. The optimization of non-crosslinking probe-AuNPs hybridization reaction. Supplementary Table 3. Results of clinical samples analysis using RT-nested multiplex-PCR, real-time RT-PCR, and CL and NCL probe-AuNPs hybridization assays. [file 12896_2021_691_MOESM1_ESM.docx]

| Hybridization temperature (ºC)  Time (min) | 80 | 85 | 90 | 95 |
| --- | --- | --- | --- | --- |
| 1 | 0.8 | 1.02 | 1.2 | 0.95 |
| 2 | 0.87 | 1.13 | 1.31 | 0.95 |
| 3 | 0.91 | 1.17 | 1.38 | 0.94 |
| 4  5 | 0.91  1.08 | 1.21  1.18 | 1.33  1.27 | 0.95  0.94 |
| Annealing temperature ( ºC) | 42 | 45 | 50 | 55 |
| Time (10 min) | 1.22 | 1.38 | 0.87 | 0.82 |

Supplementary Table 1. The optimization of cross-linking probe-AuNPs hybridization reaction.

The numbers in the table correspond to the signal-to-noise results.

Supplementary Table 2. The optimization of non-crosslinking probe-AuNPs hybridization reaction.

| Hybridization temperature (ºC)  Time (min) | 80 | 85 | 90 | 95 |
| --- | --- | --- | --- | --- |
| 1 | 1.87 | 2.4 | 2.78 | 2.93 |
| 2 | 2.07 | 2.53 | 2.9 | 2.79 |
| 3 | 2.01 | 2.67 | 3.12 | 2.84 |
| 4  5 | 2.22  2.29 | 2.51  2.63 | 3.01  3.08 | 2.63  2.33 |
| NaCl concentration (M) | 1 | 1.5 | 2 | 3 |
|  | 2.43 | 3.12 | 3.26 | 2.66 |

The numbers in the table correspond to the signal-to-noise results.

Supplementary Table 3. Results of clinical samples analysis using RT-nested multiplex-PCR, real-time RT-PCR, and CL and NCL probe-AuNPs hybridization assays

| Number of sample | RT-nested multiplex-PCR | Real-time RT-PCR | CL probe-AuNPs hybridization assay | NCL probe-AuNPs hybridization assay |
| --- | --- | --- | --- | --- |
| 1, 5-8, 12-14, 22, 27, 29, 33, 36, 39, 44, 47, 49 | Positive | Positive | Positive | Positive |
| 2-4, 9-11, 15-20, 23-26, 30-32, 34-35, 37, 40-43, 45-46, 48,50 | Negative | Negative | Negative | Negative |
| 21 | Positive | Positive | Negative | Negative |
| 28 | Positive | Positive | Positive | Negative |
| 38 | Positive | Positive | Negative | Negative |
| 35 | Negative | Negative | Negative | Positive |

CL: cross-linking; NCL: non-crosslinking
